# De-Implementation of Axillary Staging and Radiotherapy in Low-Risk Breast Cancer Patients Aged 70–79 Years from Six Italian Cancer Institutes

**DOI:** 10.3390/curroncol30040318

**Published:** 2023-04-13

**Authors:** Lauro Bucchi, Alessandra Ravaioli, Luigino Dal Maso, Fabio Falcini, Lucia Mangone, Samuele Massarut, Laura Schirosi, Anna Crispo, Patrizia Vici, Silvia Franceschi

**Affiliations:** 1 Emilia-Romagna Cancer Registry, Romagna Unit, IRCCS Istituto Romagnolo per lo Studio dei Tumori (IRST) Dino Amadori, 47014 Meldola, Forlì, Italy; 2 SOC Epidemiologia Oncologica, Centro di Riferimento Oncologico di Aviano (CRO) IRCCS, 33081 Aviano, Italy; 3 Epidemiology Unit, Azienda Unità Sanitaria Locale-IRCCS di Reggio Emilia, 42122 Reggio Emilia, Italy; 4 SOC di Chirurgia Oncologica del Seno, Centro di Riferimento Oncologico di Aviano (CRO) IRCCS, 33081 Aviano, Italy; 5 U.O.C. Anatomia Patologica, IRCCS Istituto Tumori “Giovanni Paolo II”, 70124 Bari, Italy; 6 SOC Epidemiologia e Biostatistica, Istituto Nazionale dei Tumori–IRCCS Fondazione G. Pascale, 80131 Napoli, Italy; 7 UOSD Sperimentazioni di FASE IV, IRCCS Istituto Nazionale Tumori Regina Elena, 00128 Roma, Italy; 8 Direzione Scientifica, Centro di Riferimento Oncologico di Aviano (CRO) IRCCS, 33081 Aviano, Italy

**Keywords:** axillary staging, breast cancer, de-escalation, de-implementation, guidelines, older patient, post-lumpectomy radiotherapy

## Abstract

In women aged ≥70 with low-risk breast cancer (BrC), some major international guidelines recommend against sentinel lymph node biopsy (for example, those from the Society of Surgical Oncology, U.S.) and post-lumpectomy radiotherapy (for example, those from the National Comprehensive Cancer Network, U.S.). We assessed the frequency of both procedures in six National Cancer Institutes (IRCCSs) in the North, the Centre, and the South of Italy. Data on tumour characteristics and treatment were obtained from each centre. Patients aged 70–79 years diagnosed with a pT1–pT2, clinically axillary lymph node-negative, oestrogen and/or progesterone receptor-positive, and human epidermal growth factor receptor 2-negative BrC between 2015 and 2020 were eligible for the study. Factors associated with the omission of the two procedures were evaluated using binary penalised logistic regression models. Axillary staging was omitted in 33/1000 (3.3%) women. After simultaneous adjustment for the centre of treatment and all other key variables, axillary staging was omitted more often in 2015–2016 vs. 2017–2020 (odds ratio (OR): 2.7; 95% CI: 1.0–7.5), in women aged 75–79 vs. 70–74 years (OR: 2.3; 95% CI: 1.1–4.9), and in those who had mastectomy vs. breast-conserving surgery (OR: 3.3; 95% CI: 1.2–9.0). The higher the histological grade was, the less frequent were the omissions (OR for grade 3 vs. grade 1: 0.2; 95% CI: 0.0–0.7). Post-lumpectomy radiotherapy was omitted in 56/651 (8.6%) women with no significant association with age, period, tumour stage, and tumour grade. In conclusion, the omission of axillary staging and post-lumpectomy radiotherapy in low-risk older BrC patients was rare in the Italian IRCCSs. Although women included in the study cannot be considered a nationally representative sample of BrC patients in Italy, our findings can serve as a baseline to monitor the impact of future guidelines. To do that, the recording and storage of hospital-based information should be improved.

## 1. Introduction

Patients aged 70 years or older with low-risk breast cancer (BrC), i.e., clinically axillary node-negative, oestrogen and/or progesterone receptor-positive (hormone receptor-positive, HR+) BrC, have a 10-year disease-specific survival rate of about 98% [1]. This fact notwithstanding and despite their substantial risk of toxicity and adverse medical effects, they are commonly treated as aggressively as younger patients or patients with more life-threatening subtypes of disease [2].

Axillary lymph node metastasis is the most important predictor of disease recurrence and overall survival of patients with BrC. By implication, an accurate assessment of axillary lymph node status is a critical component of the staging process [3]. In patients with early BrC, multiple randomised trials have demonstrated that breast-conserving surgery consisting of partial mastectomy followed by post-lumpectomy radiotherapy provides a long-term survival equivalent to that of patients treated with mastectomy [4]. However, axillary staging and post-lumpectomy radiotherapy are two examples of procedures in which overdiagnosis or overtreatment may take place in older women. Increasing evidence indeed suggests that the addition of axillary staging [5] and post-lumpectomy radiotherapy [1,6] to surgical excision and hormone therapy yields marginal or no prognostic improvement in older women with low-risk BrC. In particular, a meta-analysis of existing trials has confirmed that the omission of sentinel lymph node biopsy and axillary lymph node dissection does not convey an increased risk of in-breast recurrence, distant recurrence, overall mortality, or cause-specific mortality [7].

Consequently, several medical organisations and agencies currently recommend the omission of one or both procedures as a valid and safe option. The de-implementation of axillary staging, for example, is recommended by the American Society of Breast Surgeons (Columbia, MD, U.S.) [8], the American Board of Internal Medicine (Philadelphia, PA, U.S.) [9], and Ontario Health (Cancer Care Ontario, Toronto, ON, Canada) [10]. In the U.S. in particular, the National Comprehensive Cancer Network (NCCN, Plymouth Meeting, PA, USA) has recommended against radiotherapy in low-risk BrC patients aged 70 years and older since 2004 [2,11] and the Society of Surgical Oncology (SSO, Rosemont, IL, USA) against sentinel lymph node biopsy since 2016 [2].

Other medical organisations (especially in Europe) have taken a less clear-cut approach. According to the United Kingdom National Institute for Health and Care Excellence (NICE, London, UK), the omission of post-lumpectomy radiotherapy can be taken into consideration but only in the presence of clear surgical margins and a patient willingness to take adjuvant endocrine therapy for a minimum of five years [12]. The European Society of Breast Cancer Specialists (EUSOMA, Firenze, Italy) and the International Society of Geriatric Oncology (SIOG, Geneva, Switzerland) have stated that sentinel lymph node biopsy remains the standard in clinically or radiologically node-negative older women and have considered the omission of radiotherapy controversial [13]. According to the EUSOMA and SIOG, even sentinel node biopsy is associated with side effects and probably does not improve survival by itself. For this reason, the omission of axillary staging could be appropriate for frail individuals with early luminal A-like tumours. In 2021, the Italian Association of Medical Oncology (Associazione Italiana di Oncologia Medica, AIOM, Milano, Italy) has taken an intermediate approach. It has presented the omission of post-lumpectomy radiotherapy as an acceptable option and the omission of axillary staging as a merely emerging one [14].

An assessment of ‘real-world’ clinical practice in Italy is important because it would enable clinicians, firstly, to determine whether the NCCN recommendation against radiotherapy [2,11] and the SSO recommendation against sentinel lymph node biopsy [2] have had some impact on practice pattern and, secondly, to establish the baseline for possible de-escalation steps—the more so since the invitation to mammography screening is being expanded in Italy (and elsewhere) from 69 to 74 years of age [15]. Although this change is in line with the new European guidelines on breast cancer screening and diagnosis [16], it will inevitably increase the detection of indolent BrCs in elderly women.

We herein report the frequency and correlates of axillary staging and post-lumpectomy radiotherapy in low-risk BrC patients aged 70–79 years treated in six Italian Cancer Institutes from 2015 to 2020.

## 2. Materials and Methods

### 2.1. Data

The present study was conducted in six Italian National Cancer Institutes, i.e., non-university research hospitals supported by the Ministry of Health and the National Health System and denominated Istituti di Ricovero e Cura a Carattere Scientifico (IRCCSs), in the framework of Alleanza Contro il Cancro (ACC, Roma, Italy), the largest Italian network of cancer research centres. Participating IRCCS were located in the North (Aviano, Meldola, Reggio Emilia), in the Centre (Roma), and in the South of Italy (Napoli and Bari).

Data were obtained from the electronic health records available at each institution using a standardised format that contained essential information on the patient, the disease, and the treatment. Datasets were checked for logic and coding errors. When possible, missing or inconsistent information was cross-checked with the original records.

Women aged 70–79 years with a diagnosis of pT1–pT2, clinically axillary lymph node-negative, HR+, and human epidermal growth factor receptor 2 (HER2)-negative BrC between 2015 and 2020 and with available information on axillary staging and post-lumpectomy radiotherapy were eligible for the study. As far as the first endpoint is concerned, the participating centres contributed data for 1258 patients. Of these, 210 were excluded because of a lack of information on HR/HER2 status and 48 because of a lack of information on pT, leaving 1000 patients available for analysis. For the analysis addressing post-lumpectomy radiotherapy (including intraoperative radiotherapy and subsequent external beam radiation therapy), data were provided for 837 patients. Of these, 186 were excluded because of a lack of information on pT (*n* = 5), HR/HER2 status (*n* = 65), breast surgery (*n* = 84), and endocrine therapy (*n* = 32). Available patients therefore numbered 651.

In both analyses, patients who did not undergo axillary staging and patients who had sentinel lymph node biopsy as the first-line axillary staging option were assumed to be clinically node-negative. Consequently, axillary staging was defined as sentinel lymph node biopsy followed or not by lymph node dissection. HR+ BrC was defined as oestrogen and/or progesterone receptor-positive.

### 2.2. Statistical Methods

Factors potentially associated with the omission of axillary staging and post-lumpectomy radiotherapy were identified using odds ratios (ORs) and 95% confidence intervals (CIs). Centre-adjusted ORs and ORs simultaneously adjusted for all selected variables were computed using binary penalised logistic regression models, which are appropriate for risk factors with an extremely low prevalence of exposure [17].

All statistical analyses were performed using the Stata statistical package release 15.1 (StataCorp, College Station, TX, USA).

## 3. Results

As shown in Table 1, axillary staging was omitted in 33 out of the 1000 eligible patients (3.3%), and there was considerable variation between the participating centres. After adjustment for this variable, the omission of axillary staging was inversely and significantly related to high tumour grade with an OR of 0.16 (95% CI: 0.07–0.38) for grade 2 vs. grade 1 and 0.13 (95% CI: 0.03–0.55) for grade 3 vs. grade 1. After simultaneous adjustment for all variables listed in Table 1, associations also emerged with the earlier period (OR for 2015–2016 vs. 2017–2020: 2.73; 95% CI: 0.99–7.49), older age at diagnosis (OR for 75–79 vs. 70–74 years: 2.33; 95% CI: 1.10–4.94), and more aggressive surgery (OR for mastectomy vs. breast-conserving surgery: 3.25; 95% CI: 1.18–9.00). Multivariate analysis confirmed the strong effect of tumour grade.

As shown in Table 2, post-lumpectomy radiotherapy was omitted in 56 out of the 651 eligible patients (8.6%) and again showed substantial differences between the participating centres. Centre-adjusted ORs and ORs simultaneously adjusted for variables listed in Table 2 did not show any significant association with the period, patient age, tumour stage, and tumour grade, although the relationships were generally in the same direction as those found for axillary staging.

## 4. Discussion

In this Italian series of low-risk BrC patients aged 70–79 years, axillary staging was omitted in as few as 3.3% of them and post-lumpectomy radiotherapy in no more than 8.6%. It clearly appears that the conservative recommendations issued by the NCCN in 2004 [2,11] and by the SSO in 2016 have had little influence on clinical practice in Italian Cancer Institutes. The de-implementation of post-lumpectomy radiotherapy in older women with stage I HR+ BrC treated with lumpectomy and endocrine therapy also continues to face obstacles in the U.S. [2,11]. We expect to encounter comparable resistance to the new Italian guidelines released by the AIOM, which will pose a major problem of overtreatment. For this reason, we have planned to monitor their future impact on practice patterns. Concerning the omission of sentinel lymph node biopsy, the recommendation from the SSO [2] is more recent and, to the best of our knowledge, its degree of acceptance in the U.S. has not been determined yet.

We evaluated the factors that are currently and independently associated with the two procedures. Paradoxically, the omission of axillary staging was more likely before the favourable SSO recommendation of 2016 [2]. This finding is disappointing. However, a longer-term confirmation needs to be obtained. The greater prevalence of omission observed in the older 5-year age group can be interpreted as reflecting the relationship between patient age and the frequency of comorbid conditions. Finally, we hypothesise that the observed strong inverse effect of tumour grade indicates that poor or no histologic differentiation is still considered incompatible with the definition of low-risk BrC.

With respect to the omission of post-lumpectomy radiotherapy, we found no significant association with any of the abovementioned factors, although the relationships were generally in the same direction as those found for axillary staging. The absolute lack of association with patient characteristics suggests that the decision to use radiotherapy is taken a priori and can hardly be influenced by external objective factors (including new guidelines).

Barriers to the de-escalation of these breast cancer procedures of probably little value in elderly women with low-risk BrC can derive from both physicians’ and patients’ attitudes. Some radiation oncologists overestimate the risk of loco-regional recurrence in older patients if radiotherapy is not used and consider its omission a substandard treatment [18]. This is more likely to occur in Italy than in the U.S. because the recent Italian guidelines are consistent with the view that omitting radiotherapy is acceptable but not clearly recommended. Patients’ preference is often quoted to justify possible overtreatment [19] although little is known regarding patients’ understanding about pros and cons of the avoidance of a given therapy. As a potential support, web-based decision aids and other decision support systems have been developed in the field of breast cancer care to optimise, among others, the decisions on the surgical management of the axilla [20,21]. In any case, it is increasingly clear that de-implementation strategies should promote high-quality decision-making. To this end, they should be multi-level and should target the provider’s practice patterns, the patient’s beliefs, and the provider–patient communication [2]. Another proposed approach is that the role of axillary staging and post-lumpectomy radiotherapy should be discussed on a case-by-case basis in a multi-disciplinary setting to evaluate the potential impact of adjuvant treatment decisions [7].

The present study is the first product of an ACC-based research project on overdiagnosis and overtreatment of breast cancer in Italian IRCCSs. We acknowledge that it has weaknesses. One of the goals of our project is to evaluate the availability and quality of data in the IRCCSs. Although electronic databases were available in all participating centres, they were scattered in different departments and difficult to integrate because of technical and privacy-related problems. As a consequence, missing values were frequently present, also in basic variables. One of the six IRCCSs had to be excluded from the analysis of axillary staging and another one from the analysis of post-lumpectomy radiotherapy.

In addition, women included in our study cannot be considered a nationally representative sample of BrC patients in Italy. Nevertheless, we believe that our results can provide some clues about the evolution of low-risk BrC management in elderly women in Italy and will serve as a useful baseline for the monitoring of the current and future guidelines. Our findings should also stimulate the improvement in the recording and storage of hospital-based information in order to make it a valuable source of national clinical data and an essential complement to population-based cancer registries.

## Figures and Tables

**Table 1:** Factors associated with the omission of axillary staging in women aged 70–79 years with pT1–pT2, clinically axillary lymph node-negative, hormone receptor-positive, and human epidermal growth factor receptor 2-negative breast cancer from five National Cancer Institutes in Italy (2015–2020).

	Total Number	Omission of Axillary Staging		
		Number (%)	Centre-Adjusted Odds Ratio ^1^ (95% CI)	Fully Adjusted Odds Ratio ^2^ (95% CI)
All cases	1000	33 (3.3)		
IRCCS ^3^				
Romagna	414	13 (3.1)		
Bari	203	0		
Reggio Emilia	184	6 (3.3)		
Aviano	122	0		
Napoli	77	14 (18.2)		
Period				
2017–2020	678	18 (2.7)	1.00 ^4^	1.00 ^4^
2015–2016	322	15 (4.7)	2.19 (0.87–5.51)	2.73 (0.99–7.49)
Age at diagnosis (years)				
70–74	606	14 (2.3)	1.00 ^4^	1.00 ^4^
75–79	394	19 (4.8)	1.95 (0.95–3.98)	2.33 (1.10–4.94)
Surgery				
Breast-conserving	805	27 (3.4)	1.00 ^4^	1.00 ^4^
Mastectomy	175	6 (3.4)	1.57 (0.63–3.90)	3.25 (1.18–9.00)
Missing	20	0		
Tumour stage				
pT1	797	31 (3.9)	1.00 ^4^	1.00 ^4^
pT2	203	2 (1.0)	0.31 (0.08–1.14)	0.29 (0.07–1.17)
Tumour grade				
1	171	12 (7.0)	1.00 ^4^	1.00 ^4^
2	649	15 (2.3)	0.16 (0.07–0.38)	0.15 (0.06–0.38)
3	158	2 (1.3)	0.13 (0.03–0.55)	0.15 (0.03–0.67)
Missing	22	4 (18.2)		

Abbreviations: CI, confidence interval; IRCCS, Istituto di Ricovero e Cura a Carattere Scientifico (i.e., non-university research hospital supported by the Italian Ministry of Health and the National Health System). ^1^ All odds ratios were computed using binary logistic regression models with a penalised estimator. ^2^ Adjusted for all listed variables. ^3^ One of the six participating institutions was excluded from analysis due to missing data. ^4^ Reference category.

**Table 2:** Factors associated with omission of post-lumpectomy radiotherapy in women aged 70–79 years with pT1–pT2, clinically axillary lymph node-negative, hormone receptor-positive, and human epidermal growth factor receptor 2-negative breast cancer from five National Cancer Institutes in Italy (2015–2020).

	Total Number	Omission of Post-Lumpectomy Radiotherapy		
		Number (%)	Centre-Adjusted Odds Ratio ^1^ (95% CI)	Fully Adjusted Odds Ratio ^2^ (95% CI)
All cases	651	56 (8.6)		
IRCCS ^3^				
Romagna	284	16 (5.6)		
Reggio Emilia	150	15 (10.0)		
Aviano	85	5 (5.9)		
Napoli	71	1 (1.4)		
Roma	61	19 (31.1)		
Time period				
2017–2020	451	44 (9.8)	1.00 ^4^	1.00 ^4^
2015–2016	200	12 (6.0)	1.38 (0.50–3.80)	1.45 (0.52–4.03)
Age at diagnosis (years)				
70–74	404	31 (7.7)	1.00 ^4^	1.00 ^4^
75–79	247	25 (10.1)	1.29 (0.73–2.29)	1.35 (0.76–2.42)
Tumour stage				
pT1	558	47 (8.4)	1.00 ^4^	1.00 ^4^
pT2	93	9 (9.7)	1.04 (0.48–2.24)	0.99 (0.45–2.17)
Tumour grade				
1	72	6 (8.3)	1.00 ^4^	1.00 ^4^
2	451	40 (8.9)	0.84 (0.34–2.05)	0.82 (0.33–2.03)
3	111	7 (6.3)	0.42 (0.13–1.37)	0.41 (0.12–1.33)
Missing	17	3 (17.6)		

Abbreviations: CI, confidence interval; IRCCS, Istituto di Ricovero e Cura a Carattere Scientifico (i.e., non-university research hospital supported by the Italian Ministry of Health and the National Health System). ^1^ All odds ratios were computed using binary logistic regression models with a penalised estimator. ^2^ Adjusted for all listed variables. ^3^ One of the six participating institutions was excluded from the analysis due to missing data. ^4^ Reference category.

## Data Availability

Research data are stored in an institutional repository and will be shared upon request to the corresponding author.
